# Necroptosis and Neuroinflammation in Retinal Degeneration

**DOI:** 10.3389/fnins.2022.911430

**Published:** 2022-06-29

**Authors:** Yan Tao, Yusuke Murakami, Demetrios G. Vavvas, Koh-Hei Sonoda

**Affiliations:** ^1^Department of Ophthalmology, Graduate School of Medical Sciences, Kyushu University, Fukuoka, Japan; ^2^Ines and Frederick Yeatts Retinal Research Laboratory, Retina Service, Department of Ophthalmology, Massachusetts Eye and Ear, Harvard Medical School, Boston, MA, United States

**Keywords:** necroptosis, neuroinflammation, microglia, retinal degeneration, RIPK

## Abstract

Necroptosis mediates the chronic inflammatory phenotype in neurodegeneration. Receptor-interacting protein kinase (RIPK) plays a pivotal role in the induction of necroptosis in various cell types, including microglia, and it is implicated in diverse neurodegenerative diseases in the central nervous system and the retina. Targeting RIPK has been proven beneficial for alleviating both neuroinflammation and degeneration in basic/preclinical studies. In this review, we discuss the role of necroptosis in retinal degeneration, including (1) the molecular pathways involving RIPK, (2) RIPK-dependent microglial activation and necroptosis, and (3) the interactions between necroptosis and retinal neuroinflammation/degeneration. This review will contribute to a renewed focus on neuroinflammation induced by necroptosis and to the development of anti-RIPK drugs against retinal degeneration.

## Introduction

Neurodegenerative diseases are a complex group of disorders involving the processes of cell death and inflammation cell death inflammation, and include Alzheimer's and Parkinson's disease, amyotrophic lateral sclerosis (ALS) and retinal degeneration (Tansey and Goldberg, 2010; Heneka et al., 2015; Ito et al., 2016; Kauppinen et al., 2016). Inflammation is a multicellular process by which immune cells defend against pathogens and repair injury through a series of molecular and cellular changes, including the release of pro-inflammatory cytokines, apoptosis, and necroptosis (Wallach et al., 2014). Inflammation is strongly associated with cell death, especially that in a necrotic form (Wallach et al., 2014). Damage associated molecular patterns (DAMPs) generated by necrotic cells, such as interleukin-1α (IL-1α), tumor necrosis factor α (TNF-α), and high-mobility group box 1 protein (HMGB1) (Rock and Kono, 2008; Yanai et al., 2009; Wallach et al., 2014), strongly exacerbate inflammation.

Microglia are the first line of defense in the central nervous system (CNS), and exert both cytotoxic and cytoprotective effects at the crossroads between homeostasis and disease (Cherry et al., 2014; Lloyd et al., 2019; Rodríguez-Gómez et al., 2020). Persistent activation of microglia can trigger necroptosis, a type of necrosis induced by the activation of receptor-interacting protein kinase 1 (RIPK1)/RIPK3 (Weinlich et al., 2017; Galluzzi et al., 2018), and necroptosis in turn induces secretion of proinflammatory DAMPs and mediates chronic inflammation (Rodríguez-Gómez et al., 2020). It has been shown that microglial necroptosis executed by RIPK1/3 contributes to neuronal damage as well as brain regeneration (Welser et al., 2010; Ofengeim et al., 2017; He et al., 2021).

This review focuses on RIPK-dependent necroptosis which orchestrates neuroinflammation and degeneration in the CNS and retina. We also discuss the potency of anti-necroptosis therapy, which may inhibit excessive neuroinflammation and promote neuronal survival and regeneration.

## Necroptosis and Ripk Signaling

### Necroptosis—A Programmed Necrotic Cell Death

Necrosis is a type of cell death that has morphologic features of cellular swelling, cell membrane rupture and the release of intracellular molecules including DAMPs (Kaczmarek et al., 2013; Galluzzi et al., 2018; Yuan et al., 2019). Although necrosis was traditionally deemed as a passive process of cell death, it is now known that some necrosis is molecularly regulated, and differential mechanisms that underlie programmed necrosis have been identified (e.g., necroptosis, pyroptosis, and ferroptosis). Necroptosis was originally described in 2005 as necrotic cell death mediated by the activation of RIPK1 (Degterev et al., 2005). The stimuli that induce necroptosis include TNF-α (Degterev et al., 2008), Fas ligand (FasL) (Holler et al., 2000), TNF-related apoptosis-inducing ligand (TRAIL) (Holler et al., 2000), the interferons (IFNs) (Thapa et al., 2013; Dillon et al., 2014), and ligands for pathogen recognition receptors (PRRs), such as toll-like receptor 3 (TLR3) (Bermejo and Rey-Bellet, 1975), TLR4 and DNA-dependent activator of interferon regulatory factors (DAI) (Upton et al., 2012; Wang et al., 2014; Huang et al., 2015).

### Structure and Function of RIPK

**Figure 1:**
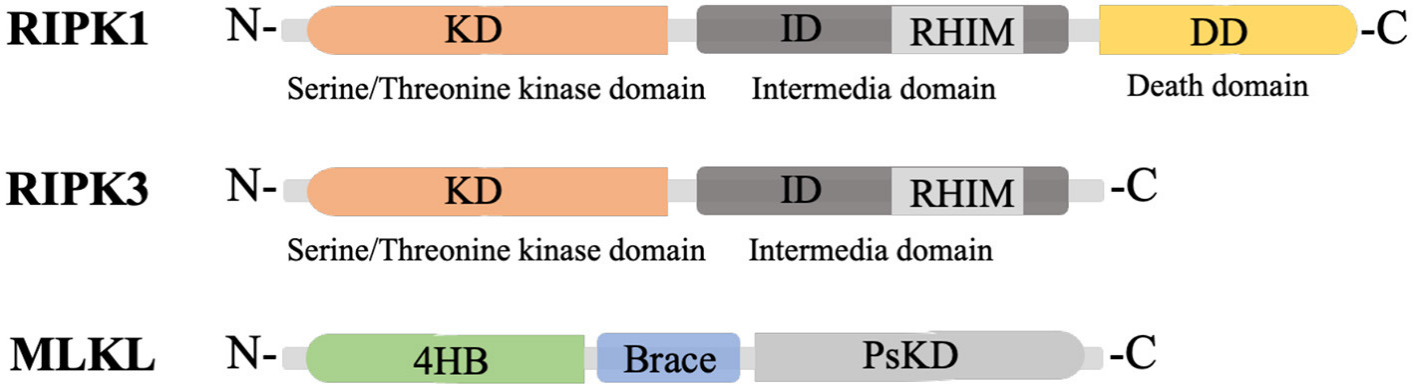
Structure of RIPK1, RIPK3 and MLKL. KD, N-terminal kinase domain; ID, intermediate domain; RHIM, RIP homotypic interaction motif; DD, death domain; 4HB, 4-helix bundle; PsKD, pseudokinase domain.

RIPK1 is a multi-functional protein that regulates cell survival, inflammation, and cell death. It is composed of three domains, an N-terminal kinase domain for phosphorylation, an intermediate domain containing an RIP homotypic interaction motif (RHIM) for RIPK1/RIPK3 interaction and pro-inflammatory action, and a C-terminal death domain (DD) for recruiting TNFα receptor 1 (TNFR1) and the TNFR1-associated death domain (TRADD) protein (Figure 1) (Yuan et al., 2019). RIPK3, a key molecule for RIPK1/RIPK3 phosphorylation, also has an N-terminal kinase domain and RHIM domain, but lacks the DD in its C-terminus (Figure 1) (Wu et al., 2014). Mixed lineage kinase domain-like protein (MLKL) consists of an N-terminal 4-helix bundle (4HB) for interacting with the membrane and the first brace helix, an intermediate brace region with two helices involved in MLKL oligomerization, and a C-terminal pseudokinase domain (PsKD) for interaction with phosphorylated RIPK3 kinase domains (Figure 1) (Murphy et al., 2013; Quarato et al., 2016; Petrie et al., 2018, 2019). MLKL forms oligomers and the 4HB insert into the plasma membrane through phosphatidylinositol phosphate (PIP)-binding sites, resulting in membrane disruption and necroptosis induction (Dondelinger et al., 2014; Su et al., 2014; Quarato et al., 2016; Petrie et al., 2019). Therefore, the phosphorylation of MLKL and the membrane translocation of MLKL oligomers are the optimal markers for necroptosis (Fricker et al., 2018).

**Figure 2:**
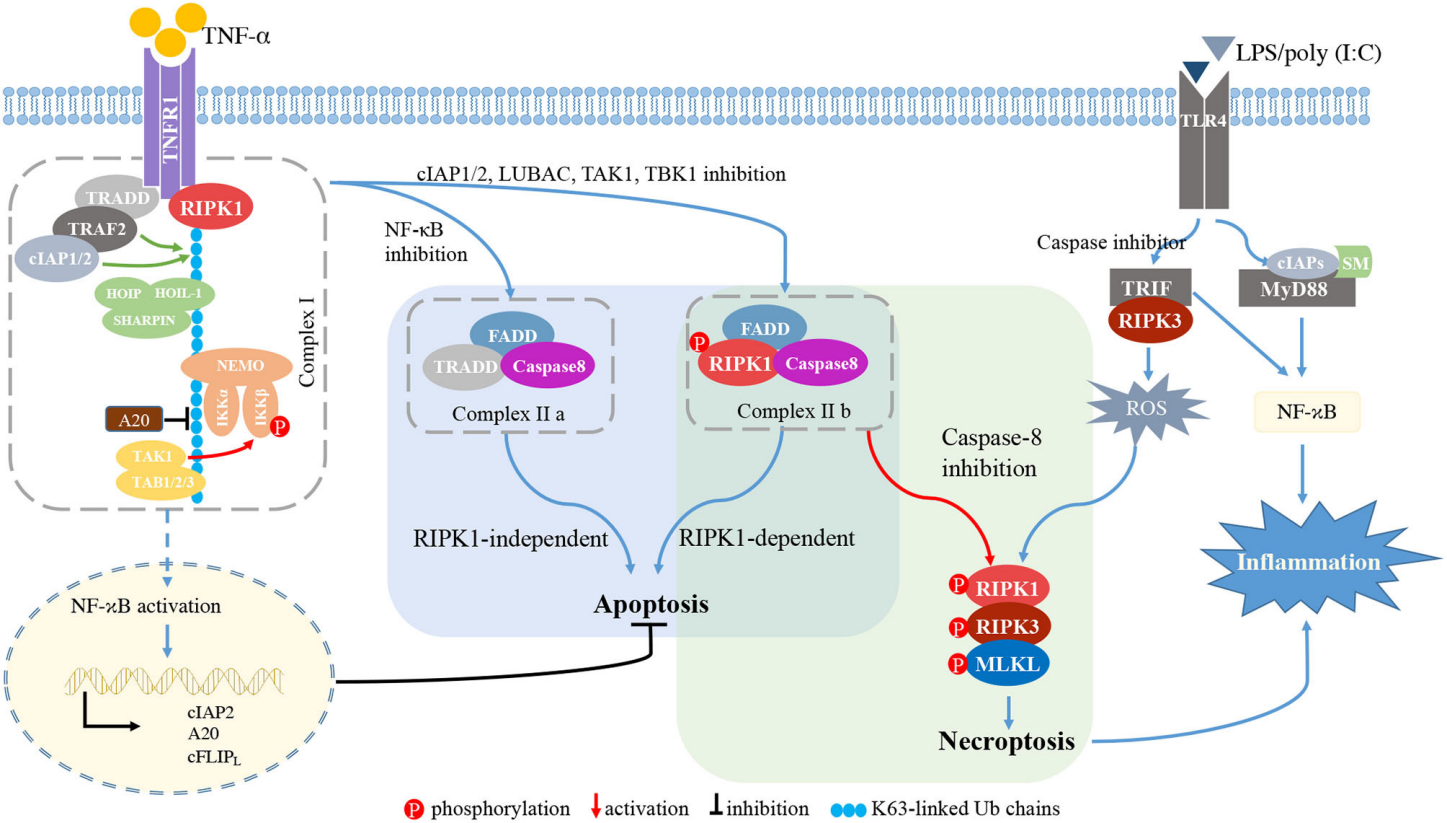
RIPK signaling. TNFR1 recruits RIPK1, TRADD, TRAF2, and cIAP1/2. cIAP1/2 initiates the ubiquitination process on complex I. The K63 Ub chain of RIPK1 links molecules in the large TNF complex I. The TAK1 complex phosphorylates IKKβ to induce NF-κB activation. When NF-κB, the complex of cIAP1/2, or LUBAC, TAK1, or TBK1 is inhibited, membrane-bound complex I is dissociated and complex II is formed. If NF-κB is inhibited, TRADD, FADD and caspase-8 form complex IIa, inducing RIPK1-independent apoptosis. When inhibition of cIAP1/2, TAK1, TBK1, or LUBAC occurs, RIPK1, FADD, caspase-8, and c-FLIPL form complex IIb, inducing RIPK1-dependent apoptosis. Procaspase-8/cFLIPL heterodimers prevent necroptosis. Upon caspase-8 inhibition, complex IIb initiates RIPK1- and RIPK3-dependent necroptosis. Activated RIPK1 heterodimerizes with RIPK3 and RIPK3 phosphorylates MLKL, thereby driving the polymerization of RIPK1, RIPK3 and MLKL. This RIPK1-RIPK3-MLKL complex is called a “necrosome.” In macrophages/microglia, LPS or poly(I:C) is recognized by TLR4 and mediates the interaction between TRIF and RIPK3. In the presence of caspase inhibitor, the TRIF/RIPK3 complex induces ROS accumulation, and subsequently triggers necroptosis independent of NF-κB activation. TLR4 also recruits MyD88 and cIAPs to activate the NF-κB pathway, thereby inducing pro-inflammatory cytokines. TNF-α, tumor necrosis factor-α; TNFR1, TNFα receptor 1; RIPK1/3, receptor-interacting protein kinases 1/3; TRADD, TNFR1-associated death domain protein; TRAF2, TNF receptor associated factor 2; cIAP1/2, cellular inhibitor of apoptosis 1/2; HOIL-1, haem-oxidized iron-regulatory protein 2 ubiquitin ligase-1; HOIP, HOIL-1 interacting protein; SHARPIN, SHANK-associated RH domain-interacting protein; NF-κB, nuclear factor-κB; NEMO, cell death-protective nuclear factor-κB essential modulator; IKKα/β, inhibitor of NF-κB kinase α/β; TAB2/3, TAK1-binding protein 2 and 3; FADD, Fas associated *via* death domain; cFLIP_L_, the long isoform of cellular FLICE-like inhibitory protein; MLKL, mixed lineage kinase domain-like protein; TIRF, TIR-domain containing adapter-inducing interferon-b; MYD88, myeloid differentiation primary response gene 88; SMs, SMAC mimetics; ROS, reactive oxygen species.

Among several stimuli for necroptosis, TNFR1 signaling is the most thoroughly investigated and proceeds as follows (Figure 2) (Seo et al., 2019). First, TNF binding to its receptor initiates the formation of complex I at the cell membrane. TNFR1 recruits RIPK1, TRADD, TNF receptor associated factor 2 (TRAF2) and cellular inhibitor of apoptosis 1/2 (cIAP1/2). cIAP1/2 initiates the ubiquitination process on the complex I (Bertrand et al., 2008; Varfolomeev et al., 2008; Annibaldi et al., 2018; Seo et al., 2019), and K63 poly-ubiquitination (Ub) of RIPK1 links many molecules, thereby forming a larger complex I. The TGF-β activated kinase 1 (TAK1) complex phosphorylates inhibitor of NF-κB kinase β (IKKβ) to activate the nuclear factor-κB (NF-κB) pathway (Jha et al., 2019; Seo et al., 2019). The NF-κB-dependent production of pro-survival genes, such as cIAP1, A20 and caspase-8-like inhibitory protein (c-FLIP_L_), protects cells from RIPK1-independent death. A20 is a deubiquitinating enzyme that prevents NF-κB activation and restricts TNF-induced apoptosis (Onizawa et al., 2015). cIAP1 and c-FLIP_L_ facilitate NF-κB activation and inhibit necroptosis. NF-κB activation also modulates immune response *via* the production of proinflammatory molecules (Jha et al., 2019; Jensen et al., 2020).

The TNFR1-mediated signaling pathway can induce apoptosis. Once the NF-κB pathway or complex I is inhibited by cIAP1/2 or by the linear ubiquitination assembly complex (LUBAC), TAK1, or TANK binding kinase 1 (TBK1), the membrane-bound complex I is dissociated and cytosolic complex IIa and IIb are formed. TRADD, FADD and caspase-8 form complex IIa and mediate RIPK1-independent apoptosis (Dondelinger et al., 2016; Annibaldi and Meier, 2018). Complex IIb is composed of RIPK1, FADD, caspase-8 and c-FLIP_L_, and initiates RIPK1-dependent apoptosis (Cho et al., 2009; Oberst et al., 2011; Sun et al., 2012).

Upon caspase-8 inhibition, complex IIa contributes to necroptotic induction by recruiting RIPK1, and complex IIb initiates RIPK1- and RIPK3-dependent necroptosis. Activated RIPK1 heterodimerizes with RIPK3 and RIPK3 phosphorylates MLKL, thereby polymerizing RIPK1, RIPK3 and MLKL (Sun et al., 2012; Zhao et al., 2012). This RIPK1-RIPK3-MLKL complex, called a “necrosome,” triggers the subsequent cell lysis. A20 inhibits formation of the RIPK1-RIPK3 complexes by deubiquitinating RIPK3 (Onizawa et al., 2015; Galluzzi et al., 2018).

The PRR family can also initiate necroptosis. TLR4 recruits the adaptor molecules myeloid differentiation primary response gene 88 (MyD88) and TIR-domain containing adapter-inducing interferon-b (TRIF), while TRIF is the sole adaptor molecule for TLR3 (Kawai and Akira, 2010). Stimulation of TLR4/TLR3 *via* LPS or poly(I:C) induces the formation of the TRIF-RIPK3 complex (Kaiser and Offermann, 2005; He et al., 2011). In the presence of caspase-8 inhibitor, reactive oxygen species (ROS) accumulation occurs downstream of the TRIF-RIPK3 complex, and the subsequent c-jun N-terminal kinase (JNK) activation forms the RIPK1-RIPK3-MLKL complex and induces necroptosis independent of NF-κB activation (He et al., 2011; Kim and Li, 2013). TLR4 also recruits the adaptor molecules MyD88 and cIAPs to induce the NF-κB pathway activation and release of pro-inflammatory cytokines (Figure 2).

## Interactions Between Necroptosis and Neuroinflammation

Necroptosis can trigger inflammation. Activated microglia/macrophages also induce necroptosis at the chronic inflammatory site, thereby forming a positive feedback loop. In neurodegenerative diseases, dying or dead neuronal cells release DAMPs and activate PRRs (Wallach et al., 2014). PRR signaling activates microglia and transforms them to a pro-inflammatory phenotype that produces abundant pro-inflammatory cytokines (Fernández-Velasco et al., 2014). TNF-α and PRPs-mediated signals activate intracellular pathways, such as the NF-κB activation pathway, which leads to the transcription of various pro-inflammatory molecules, and the RIPK pathway, which mediates microglial activation and necroptosis (Granger and Kolb, 1968; Liu et al., 2017).

### Characteristics of Microglial Activation in Neurodegeneration

Microglia are resident macrophages in the CNS and retina that surveil the surrounding environment and maintain tissue homeostasis (Nimmerjahn et al., 2005). Microglia can dynamically change their morphology and function in response to microenvironmental alterations (Choi et al., 2021). Although, in healthy individuals, blood circulating monocytes cannot enter the CNS and retina due to the healthy blood-brain and retina-barrier, under disease conditions they can infiltrate and differentiate into macrophages.

Microglia exhibit diverse functional phenotypes depending on the disease context (Ransohoff, 2016). In the same way that peripheral macrophages are traditionally classified into two major phenotypes (Martinez and Gordon, 2014) (i.e., M1 and M2), microglia can also polarize into both pro- and anti-inflammatory states at the diseased loci. Once microglia and/or macrophages detect DAMPs, they convert to the pro-inflammatory M1-like phenotype and form a high motility amoeboid shape (Chen and Xu, 2015). Activated microglia translocate to the damage site and secrete pro-inflammatory cytokines (IL-1β, IL-6, IL-8, TNF-α, iNOS) (Kalkman and Feuerbach, 2016; Aguzzi and Zhu, 2017). After the damaging molecules are diminished, the microglia change their polarity to an anti-inflammatory M2-like phenotype, improving their phagocytotic function, secreting IL-4, IL-10, IL-13, TGF-β, and arginase 1 and clearing the cellular debris to promote recovery (Cherry et al., 2014; Park et al., 2016). Triggering-receptors-expressed-on-myeloid-cells 2 (TREM2) is one kind of microglial receptor that is highly expressed in anti-inflammatory or pro-regenerative microglia and involved in phagocytosis and immune regulation (Paloneva et al., 2002; Hickman and El Khoury, 2014; Hickman et al., 2018). Overexpression of TREM2 enhances phagocytic activity and down-regulates pro-inflammatory responses (Han et al., 2017). In contrast, deletion of TREM2 or the adaptor tyrosine kinase-binding protein (TyroBP or DAP12), which binds to TREM2, leads to an excess of the pro-inflammatory phenotype, which decreases microglial survival and causes amyloid plaque deposition in experimental models of Alzheimer's disease (AD) (Ito and Hamerman, 2012). Therefore, these findings suggest that TREM2 is a key immune regulator that mediates the homeostatic function of microglia. However, it should be noted that there are more diverse and complex phenotypes in microglia and macrophages, and their plasticity (reaction states) and diversity (subtypes) should both be considered rather than merely the two commonly phenotypes (Stratoulias et al., 2019). Single-cell level analyses will help further clarify the microglia/macrophage subsets that regulate CNS health and diseases (Ransohoff, 2016; Hammond et al., 2019; Masuda et al., 2019; Stratoulias et al., 2019).

### RIPK Modulates Microglial Activation and Necroptosis in Neuroinflammation

Microglia recognize the DAMPs through pattern recognition proteins (PRPs) such as TLRs. As shown in Section 2.2, TLR signaling mediates microglial activation (i.e., M1-like polarization) as well as apoptosis/necroptosis through RIPK recruitment into the cell death complex. TNF-α released from microglia may also activate RIPK pathways in the surrounding neuronal cells as well as microglia themselves.

Soluble oligomeric amyloid β (Aβ) has been shown to stimulate TNF-α release from activated microglia, leading to the induction of neuronal necroptosis in an *in vitro* model of AD (Salvadores et al., 2021). *Ripk*3^−/−^ and *Mlkl*^−/−^ mice favor pro- to anti-inflammatory phenotype transformation of microglia in response to ischemic cortex injury (Yang et al., 2018). Therefore, RIPK may regulate necroptosis and microglia/macrophage polarization in neurodegeneration (Table 1).

**Table 1:** Pharmacological and genetic interventions that manage cell death and inflammation in RIPK-related pathways in CNS diseases.

**Role of the RIPK/related pathway**	**Disease and model**	**Mode of RIPK inhibition**	**Outcome**	**References**
Mediates DAM response and reduces the lysosomal function of microglia	AD (APP/PS1)	RIPK1 inhibitor; *Ripk1-D138N* double mutant mice	• Attenuated DAM response •Enhanced phagocytosis of Aβ	Ofengeim et al., 2017
Activates the NLRP3 inflammasome	Bacterial encephalitis (LPS treatment in macrophages)	*Ripk3* knockout	• Inhibited the IL-1 secretion induced by LPS and IAP antagonist	Vince et al., 2012
Induces microglial necroptosis	ICH (injection of autologous blood from the femoral artery into the right basal ganglia)	Melatonin (upregulation of deubiquitinating enzyme A20)	• Reduced microglial necroptosis and TNF secretion • Suppressed neuronal necroptosis	Lu et al., 2019
Induces necroptosis and age-related neuroinflammation	Aging (old WT mice)	RIPK1 inhibitor; *Mlkl* knockout; *Ripk3* knockout	• Reduced age-associated neuroinflammation	Thadathil et al., 2021
Promotes axonal degeneration and neuroinflammation	ALS (Optn-deficient mice, SOD1 transgenic mice)	RIPK1 inhibitor; *Ripk1-*D138N mutant	• Suppressed neuronal cell death • Reduced microglial response	Ito et al., 2016

*DAM, disease-associated microglia; AD, Alzheimer's disease; APP/PS1, amyloid precursor protein/presenilin 1; Aβ, amyloid-β; ALS, amyotrophic lateral sclerosis; ICH, intracerebral hemorrhage*.

RIPK may contribute to microglia/macrophage polarization *via* a mechanism that is at least partly independent of necroptosis (Ofengeim and Yuan, 2013; Ofengeim et al., 2015, 2017; Kondylis et al., 2017; Ueta et al., 2019). Our previous study showed that RIPK inhibition suppresses M2-like polarization through caspase activation and attenuates the formation of laser-induced choroidal neovascularization (CNV) (Ueta et al., 2019). Both *in vivo* and *in vitro* experiments have shown that infiltrating macrophages, rather than vascular endothelial cells, are the main target for catalytic inhibition of RIPK. These findings suggest that RIPK has a non-necrotic function in angiogenesis *via* regulation of the macrophage phenotype. Ofengeim et al. reported a non-cell death function of RIPK1—namely, mediation of the disease-associated microglia (DAM) phenotype—in AD pathology (Ofengeim et al., 2017). They showed that RIPK1 is highly expressed in the cerebral microglia of patients with AD. In a mouse model of AD and *in vitro* cultured microglia, RIPK1 inhibition was shown to attenuate the DAM response and to enhance phagocyte activity to clear Aβ (Ofengeim et al., 2017). Transcriptional analysis found that RIPK1 induces Cst7 expression, which regulates the lysosomal function of microglia, suggesting that RIPK1-dependent transcription disturbs the homeostatic function of microglia and leads to Aβ accumulation in AD (Ofengeim et al., 2017). Alternatively, Vince et al. demonstrated that RIPK3 directly regulates NLRP3 inflammasome activation and IL-1β secretion in macrophages (Vince et al., 2012). They showed that RIPK3 mediates IAP-antagonist-induced IL-1β secretion prior to inducing necrotic changes (Vince et al., 2012).

### Ubiquitination in Microglia-Related Inflammation

Ubiquitin participates in various biological processes, including protein degradation, transcription, DNA and immune regulation (Husnjak and Dikic, 2012; Zinngrebe et al., 2014). Several E3 ubiquitin ligases are involved in the regulation of MyD88- and TRIF-dependent signaling from TLRs (Wertz and Dixit, 2010). TRAF6, one of the ubiquitin E3 ligases, mediates K63-linked ubiquitination of the IKK complex subunit, IKKγ, and activates MyD88- and TRIF-dependent NF-κB signaling (Walsh et al., 2008). A20 can modulate ubiquitination and remove K63 polyubiquitin chains from target proteins to terminate signaling, as shown in Figure 2 (Komander et al., 2009; Mohebiany et al., 2020).

Kinsella et al. (2016) identified that the Bcl-2 family protein BH3-interacting domain death agonist (Bid) strengthens the TLR4-NF-κB pro-inflammatory response by promoting K63-linked polyubiquitination of TRAF6 in microglia. In a subsequent study, they demonstrated that Bid modulates MyD88- and TRIF-dependent signaling by attenuating the cleavage of polyubiquitin chains, thereby enhancing the inflammatory response (Kinsella et al., 2018).

A study on AD demonstrated that the E3 ubiquitin ligase COP1 inhibits the activation of microglia and the release of pro-inflammatory factors by degrading the transcription factor CCAAT/enhancer binding protein beta (c/EBPβ) (Ndoja et al., 2020). These transcription factors promote gene expressions related to microglial activation and inflammation. Increased secretion of pro-inflammatory factors and neurotoxicity in COP1-deficient microglia were observed in a mouse model of tau-mediated neurodegeneration and microglia-neuronal co-cultures. Thus, COP1 is important for suppression of the pathogenic c/EBPβ-dependent gene expression process in microglia.

RIPK is also involved in K63-linked Ub. IAPs are the key E3 ubiquitin ligases that ubiquitinate RIPK1/2 and cause NF-κB activation (Figure 2) (Jensen et al., 2020). Several studies have shown that RIPK1-mediated inflammatory signaling can be inhibited by second mitochondria-derived activator of caspases (SMAC), a small-molecule antagonist of IAPs (Busca et al., 2018; Goncharov et al., 2018). SMAC mimetics can also suppress the pro-inflammatory response in TLR signaling (Figure 2) (Tseng et al., 2010). Deubiquitinating enzyme is an alternative target to modulate RIPK activation. Lu et al. recently demonstrated that microglial necroptosis is suppressed by melatonin *via* the regulation of A20 in a model of intracerebral hemorrhage (Lu et al., 2019). Therefore, targeting ubiquitination may be a promising anti-inflammatory strategy.

### The Role of Microglial Necroptosis in Neuroinflammation

Necroptosis can occur in neurons as well as microglial cells in CNS diseases. Thadathil et al. investigated the expression of p-MLKL, a marker of necroptosis, in the brains of young and aged mice and showed that nearly 70–80% of p-MLKL immunoreactivity is observed in neurons and <10% in microglia (Thadathil et al., 2021). Blocking or inhibiting necroptosis resulted in a significant reduction in neuroinflammation in aged mice (Thadathil et al., 2021). Therefore, necroptosis may be critically important in age-associated neuroinflammation and neurodegeneration.

Ito et al. investigated the functional roles of RIPK in an amyotrophic lateral sclerosis (ALS) model. Mutations in the optineurin *OPTN* gene have been implicated in patients with ALS (Ito et al., 2016). Using an ALS model of *optineurin* (*Optn*)-deficient mice, they reported that RIPK1 inhibition reduces the levels of proinflammatory cytokines, including IL-1α, IL-1β, interferon-γ (IFNγ), and TNF-α, in the spinal cord (Ito et al., 2016). RNA-sequencing analysis of *Optn*^−/−^, and *Optn*^−/−^*; Ripk1*^*D*138*N*/*D*138*N*^ microglia revealed that the M1-like phenotype in *Optn*^−/−^ microglia is suppressed by RIPK1 inhibition (Kigerl et al., 2009; Ito et al., 2016). In addition, necrotic cell death in the spinal cord of *Optn*^−/−^ mice was rescued by RIPK inhibition, which led to improved motor function (Ito et al., 2016). In pathological spinal cord sections from patients with ALS, increased co-immunostainings of RIPK1, RIPK3, MLKL, RIPK1 p-Ser14/15, p-MLKL, and microglia were observed (Ito et al., 2016). Therefore, RIPK1 may be a critical mediator of microglial activation as well as necroptosis in the axonal pathology of ALS.

## The Role of Ripk in Retinal Degeneration and Neuroinflammation

**Figure 3:**
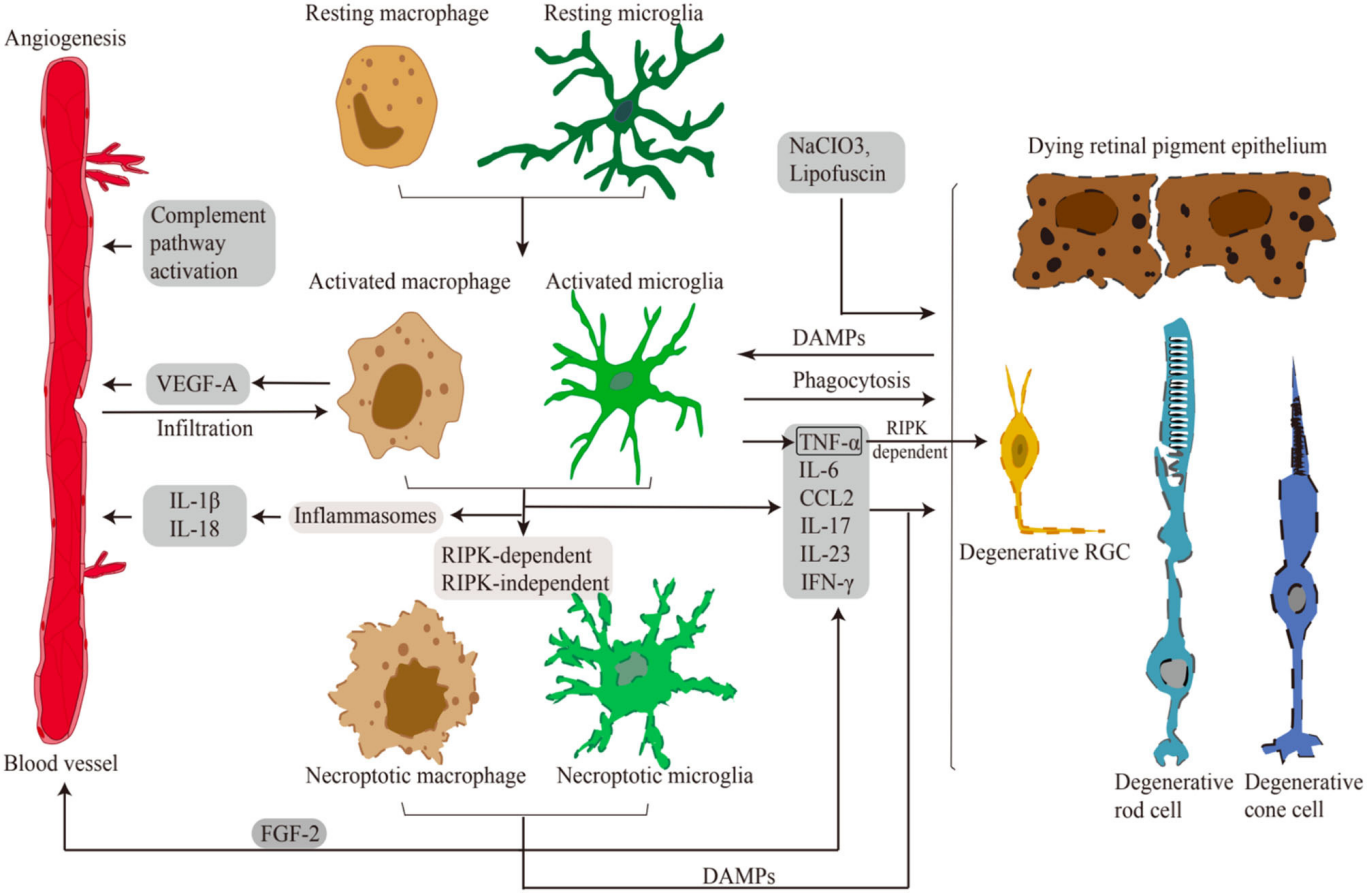
Microglia/Macrophages in retinal disorders. Schematic representation of necroptotic and activated microglia/macrophages in retinal necroptosis and inflammation. Various damages and DAMPs activate innate immune cells. Activated and necroptotic microglia/macrophages *via* inflammasome-dependent/-independent mechanisms secrete inflammatory and angiogenic mediators. These pro-inflammatory and angiogenic factors induce choroidal neovascularization. Pro-inflammatory factors and DAMPs also lead to necrotic cell death of RGCs, RPE cells and photoreceptors. Moreover, activated microglia/macrophages infiltrate to the outer retina to phagocytose dead cells and modulate inflammation.

Given the significant roles of the RIPK pathway in necroptosis induction and microglial activation in CNS disorders, it would be natural to expect that RIPK is involved in retinal necroptosis and inflammation. Indeed, although it was traditionally considered that apoptosis is the main form of cell death in retinal degeneration, our group revealed that RIPK-dependent necroptosis is redundantly activated when caspases are inhibited in experimental retinal detachment (RD) (Trichonas et al., 2010). Rosenbaum et al. also demonstrated that treatment with Nec-1 prevents retinal cell death in a rat model of retinal ischemia (Rosenbaum et al., 2010). It is now known that RIPK plays pivotal roles in various retinal and optic nerve disorders, including inherited retinal diseases (IRDs), age-related macular degeneration (AMD), and glaucoma, even in the absence of caspase inhibition (Trichonas et al., 2010; Murakami et al., 2012, 2014; Kataoka et al., 2015; Do et al., 2017; Kayama et al., 2018). In the discussion below, we introduce studies addressing the RIPK function in necroptosis, microglial activation, and neuroinflammation in retinal degeneration (Figure 3; Table 2).

**Table 2:** Pharmacological and genetic interventions that manage cell death and inflammation in RIPK-related pathways in retinal diseases.

**Role of the RIPK-related pathways**	**Disease and model**	**Mode of RIPK inhibition**	**Outcome**	**References**
Mediates photoreceptor necroptosis and neuroinflammation	RD (subretinal injection of sodium hyaluronate), RP (rd10, rd1), acute retinal neural injury (NMDA-damaged mice), achromatopsia (*pde*6*c*^w59^ mutant zebrafish)	RIPK inhibitors; *Ripk3* knockout; *Ripk3* knockdown	• Rescued photoreceptor cells • Reduced Iba1^+^ or CD11b^+.^ microglia/macrophages	Trichonas et al., 2010; Murakami et al., 2012; Viringipurampeer et al., 2014; Huang et al., 2018
Activates the NLRP3 inflammasome	RD (subretinal injection of sodium hyaluronate)	*Ripk3* knockout	• Inhibited NLRP3/ IL-1β secretion • Rescued photoreceptor cell death	Kataoka et al., 2015
Mediates RPE necroptosis and DAMPs-mediated neuroinflammation	Dry AMD (dsRNA-induced retinal degeneration)	RIPK1 inhibitor; *Ripk3* knockout	• Rescued RPE and photoreceptor cell death • Reduced release of DAMPs and attenuated microglia/macrophages activation	Murakami et al., 2014
Induces microglial necroptosis	RP (rd1), acute retinal neural injury (NMDA-damaged mice)	RIPK1 inhibitor	• Decreased cytokine production • Rescued photoreceptor and retinal ganglion cell death	Huang et al., 2018
Induces microglial necroptosis	Retinal neovascularization (OIR)	RIPK1 inhibitor; Microglia-specific *Ripk3* knockout	• Decreased FGF2 release • Suppressed angiogenic retinopathy	He et al., 2021
Activates macrophages (M2 polarization)	Wet AMD (laser CNV)	RIPK1 inhibitor; *Ripk1* K45A mutant	• Suppressed M2-like polarization of macrophages • Attenuated pathological angiogenesis	Ueta et al., 2019
Induces necroptosis and neuroinflammation	Glaucoma (IR, acute high intraocular pressure)	RIPK1 inhibitor	• Reduced retinal damage and neuroinflammation	Rosenbaum et al., 2010; Dvoriantchikova et al., 2014
Induces RGCs necroptosis	Glaucoma (ON, optic nerve crush model)	RIPK1 inhibitor; *Ripk3* knockout	• Promoted RGC survival and axon regeneration	Kayama et al., 2018

*RD, retinal detachment; AIF, apoptosis-inducing factor; RP, retinitis pigmentosa; DR, diabetic retinopathy; AMD, age-related macular degeneration; OIR, oxygen-induced retinopathy model; RGCs, retinal ganglion cells; IR, ischemia–reperfusion*.

### Role of RIPK in Retinal Detachment

Photoreceptor cells receive metabolic support from the underlying retinal pigment epithelium (RPE) and choroidal vessels, and when the neuroretina is physically detached from the RPE, photoreceptor cells start to die. Experimental models of RD induced by the subretinal injection of sodium hyaluronate and human samples with RD have shown that photoreceptor cell death is induced as early as 12 h and peaks at around 2–3 days after RD (Cook et al., 1995; Hisatomi et al., 2001; Arroyo et al., 2005). Hypoxia plays an important role in this process, because oxygen therapy substantially prevents photoreceptor cell loss in a cat model of RD (Lewis et al., 2004). Neuroinflammation also contributes to RD-induced retinal degeneration. Multiple cytokines/chemokines, including TNF-α, IL-1β, and MCP-1, are substantially elevated in the eyes in both experimental and human RD (Nakazawa et al., 2006a; Yoshimura et al., 2009). Moreover, genetic knockout of *Mcp-1* attenuates macrophage infiltration and prevents photoreceptor cell death in a rodent model of RD (Nakazawa et al., 2007). Sweigard et al. suggested that hypoxia mediates the activation of the alternative complement pathway in the detached mouse retina, indicating a molecular link between hypoxia and neuroinflammation (Sweigard et al., 2015).

Apoptosis is the main form of photoreceptor cell death after RD (Cook et al., 1995; Hisatomi et al., 2001; Arroyo et al., 2005). Consistent with the apoptotic morphological changes, such as pyknosis, both caspase activation in intrinsic and extrinsic pathways and upregulated expression of death receptor ligands (e.g., TNF-α and Fas-L) are observed in the rat retina after RD (Zacks et al., 2003, 2004). Paradoxically, however, caspase inhibition *via* intravitreal injection of pan-caspase inhibitor did not lead to protection against RD-induced retinal degeneration, suggesting the presence of other or redundant effectors for the photoreceptor cell death after RD.

As described above, caspases and RIPK redundantly function as cell death effectors in TNF-α signaling. Given the evidence that RIPK-dependent necrosis is strongly induced when the caspase pathway is blocked under various physiological and pathological conditions, we hypothesized that RIPK may mediate photoreceptor cell death after RD in concert with caspases. Indeed, we found that caspase inhibition in rodent models of RD substantially reduced apoptosis of photoreceptor cells while simultaneously increasing necrotic cell death in the detached retina. This necrotic change was rescued by additional treatment with Nec-1 or *Ripk3* deficiency, indicating that photoreceptor cell death after RD is redundantly regulated by at least two cell death pathways, i.e., caspase-dependent apoptosis and RIPK-dependent necroptosis (Trichonas et al., 2010). Consistent with our findings, Dong et al. demonstrated that Nec-1 inhibits RIPK phosphorylation and prevents necrotic photoreceptor cells after experimental RD (Dong et al., 2012).

RIPK inhibition in experimental RD prevents photoreceptor cell death, in addition to attenuating the infiltration of CD11b-positive microglia/macrophages in the detached retina (Trichonas et al., 2010). Kataoka et al. further investigated the roles of RIPK in inducing neuroinflammation after mouse experimental and human RD and showed that RIPK regulates NLRP3/caspase-1 inflammasome activation and prevents IL-1β secretion from subretinal microglia/macrophages (Kataoka et al., 2015). Because Vince et al. (2012) demonstrated that RIPK3 directly promotes IL-1β secretion in macrophages *via* inflammasome-dependent and -independent mechanisms, it may be possible that RIPK directly modulates microglia/macrophage activation after RD, in addition to indirectly activating neuroinflammation through the release of necrotic DAMPs.

### Role of RIPK in Retinitis Pigmentosa

Retinitis pigmentosa (RP) is a set of hereditary retinal diseases characterized by the degeneration of rod and cone photoreceptors with genetic defects (Hartong et al., 2006). The typical progression in patients with RP begins with night blindness and loss of mid-peripheral visual field due to rod cell dysfunction and death, followed by loss of peripheral and central vision due to cone cell death (Hartong et al., 2006; Murakami et al., 2015). Many studies have shown that multiple signaling pathways lead to photoreceptor death, including apoptosis, autophagy and necroptosis (Murakami et al., 2012, 2015; Sizova et al., 2014; Athanasiou et al., 2018; Viringipurampeer et al., 2019). However, the specific mechanisms predominating in each of rod and cone photoreceptor cell death in RP remain elusive.

Rod cell death in RP is induced by mutations in the causal genes, most of which are related to rod function, structure or homeostasis, and the dead cells exhibit apoptotic morphology. In contrast, cone cells, which do not express deleterious genes, remain healthy in the early stage of disease, but also degenerate after rod cell death. The mechanisms of this secondary cone cell death have not been fully understood; however, accumulating evidence suggests that microenvironmental alterations such as neuroinflammation, oxidation and metabolic imbalance contribute to the cone cell death in RP (Yoshida et al., 2013; Olivares-González et al., 2018, 2021; Newton and Megaw, 2020). Interestingly, morphological analyses of the postmortem eyes of RP patients demonstrated some necrotic features, such as swollen cytoplasm and membrane rupture in remaining cones (To et al., 2004). Consistent with these anatomical features, our group demonstrated that RIPK is essential for induction of cone necroptosis and microglia activation in rd10 mice, a mouse model of RP induced by a missense mutation in the *Pde6*β gene, and both effects could be suppressed by *Ripk3* deletion (Murakami et al., 2012). Subsequently, we found significantly enlarged cone cells in the macula of human RP patients using adaptive optics technology, and observed higher levels of HMGB1, a DAMP released from necrotic cells, in the vitreous of RP patients (Scaffidi et al., 2002; Murakami et al., 2015).

Consistent with our findings, Viringipurampeer et al. (2014) identified that cones expressed high levels of RIPK1 and RIPK3, and that the dying cones could be rescued by knockdown of *Ripk3* in *pde6c*^*w*59^ mutant zebrafish. Yang et al. (2017) also revealed that RIPK1 and RIPK3 were markedly upregulated in the retinas of sigma-1 receptor (S1R)-deficient rd10 mice, accompanied with a deteriorated loss of cones. A recent study reported that the necroptosis pathway is highly activated during photoreceptor death in Pro23His (P23H) transgenic albino rats, and P23H is also the most common mutation in autosomal dominant RP (Kakavand et al., 2020). Increased phospho-MLKL has been observed in the inner and outer segments of the P23H-3 mouse retina (Kakavand et al., 2020). Furthermore, Sato et al. (2013) observed TNF-induced rod and cone necroptosis in *Irbp*-deficient mice. Interphotoreceptor retinoid-binding protein (IRBP) secreted by photoreceptors is important for photoreceptor survival, and *IRBP* mutation is associated with human RP.

Therefore, RIPK-mediated necroptosis may be a common mechanism for cone cell death in RP, and RIPK-mediated necroptosis may be a novel target to prevent the death of photoreceptors in RP patients.

Cell death and inflammation are interconnected. Indeed, we previously demonstrated that microglial activation is suppressed by *Ripk3* deficiency in rd10 mice. This anti-inflammatory effect of RIPK inhibition may be explained by the reduced release of DAMPs from necrotic cone cells as well as by the attenuation of microglial activation/necroptosis.

To our knowledge, only a single report has described the implications of microglial necroptosis in both *in vitro* and *in vivo* (rd1 mice, pde6β^rd1^ and NMDA-damaged mice) models of retinal degeneration (Huang et al., 2018). TLR4 is involved in the activation of microglia in these models, in association with infiltration of Iba-1^+^/RIPK3^+^ necroptotic microglia at the site of degeneration. These necroptotic microglia secrete inflammation mediators, e.g., TNF-α, chemokine (C-C motif) ligand 2 (CCL2), IL-17, IL-23 and IFN-γ. Pharmacological blockade of RIPK1 has been shown to ameliorate microglial activation and retinal degeneration in both an NMDA-induced retinal neural injury model and murine BV2 microglial cells (Huang et al., 2018). These findings suggest that microglial necroptosis may regulate neuroinflammation in RP, at least in part.

### Role of RIPK in Dry Age-Related Macular Degeneration

AMD is a primary cause of central visual loss in developed countries and affects primarily the elderly (Smith et al., 2001). Early AMD is characterized by pathological deposits (drusen) between the RPE and the Bruch's membrane (Al-Zamil and Yassin, 2017; Handa et al., 2019). RPE are monolayer cells maintaining the functionality of overlying photoreceptors (Boulton and Dayhaw-Barker, 2001). Widespread drusen, various pathologies such as inflammation, and various genetic and environmental factors promote RPE dysfunction and photoreceptor death (Handa et al., 2019; Miller et al., 2021). Advanced late-stage dry AMD, also known as geographic atrophy (GA), is associated with the degradation of RPE cells in a non-neovascular form (Handa et al., 2019; Miller et al., 2021). Oxidative stress is implicated in the pathogenesis of both wet and dry AMD, and it can cause blood-retinal barrier breakdown that triggers chronic inflammation (Barnett and Handa, 2013).

Alterations of many inflammatory cytokines have been detected in plasma, serum or intraocular fluid in patients with AMD, but regrettably there is no stable trend or consistency in the reported findings (Tan et al., 2020).

Infiltration of macrophages has been detected in late advanced dry AMD and neovascular AMD (Penfold et al., 1985; Lad et al., 2015). Activated immune cells such as microglia/macrophages can migrate or infiltrate to the outer nuclear layer and remove photoreceptor debris, which is believed to be related to atrophic AMD (Killingsworth et al., 1990; Gupta et al., 2003). Analysis of a single-cell transcriptomic atlas of human retinas indicated that microglia are one of the factors most predictive of AMD, which highlights the importance of microglia in AMD pathogenesis (Menon et al., 2019). A histological study of the human eyes revealed that higher numbers of macrophages are concentrated in the choroid in late dry AMD than in normal AMD (Wang et al., 2022). Taken together, these data indicate a pathogenetic role of inflammation and immune response in AMD. Yet there is still controversy and debate regarding the origin of the cells reported in histopathology as macrophages, with some studies supporting the notion that some of these cells may not be bona-fide immune cells but rather transdifferentiated RPE cells (Lad et al., 2015; Curcio and Ach, 2016).

The RIPK-dependent necrotic pathway is crucial in RPE and photoreceptor cell death in AMD. Our previous study identified that programmed necroptosis is the main mechanism for cell death of RPE in dsRNA-induced retinal degeneration, a model of dry AMD (Murakami et al., 2014). *Ripk3* deficiency in this model reduced the release of DAMPs and suppressed the inflammatory response in the retina (Murakami et al., 2014). These findings suggest that RIPK-dependent necroptosis amplifies neuroinflammation by regulating the release of intracellular DAMPs (Murakami et al., 2014). Oxidative stress is one important component of AMD pathogenesis. Hanus et al. (2015) confirmed that RPE necroptosis is the predominant mechanism of NaIO_3_-induced RPE cell death, a model of dry AMD induced by oxidative injury. Because caspase-8 is severely downregulated in the mature RPE (Yang et al., 2007), RPE cells may be predisposed to undergo necroptosis under a stressed condition.

A study by Pan et al. (2021) reported that retinal lipofuscin activates an atypical necroptosis in RPE cells as well as macrophages/microglia. That study provided new insights into necroptosis activation in aged human retinas with AMD. Interestingly, its authors demonstrated an atypical RPE necroptotic mechanism that involves neither RIPK1 nor RIPK3, but rather is mediated by lysosomal membrane permeabilization (LMP) and subsequent MLKL phosphorylation (Pan et al., 2021). In addition to RPE cells, phospho-MLKL staining was also observed in microglia/macrophages, which phagocytosed the sloughed RPE fragments (Pan et al., 2021). These findings suggest the intimate interactions between necroptosis and neuroinflammation in the pathology of dry AMD. They also show that anti-necroptosis agents that target MLKL phosphorylation (Nec-7) and oligomerization (NSA) prevent light-independent lipofuscin-elicited necroptosis. Thus, targeting lipofuscin-induced necroptosis may be a new therapeutic strategy for dry AMD.

### Role of RIPK in Wet Age-Related Macular Degeneration

CNV is the end stage of the wet form of AMD, with growth of new blood vessels between Bruch's membrane and the RPE (Hobbs and Pierce, 2021). After CNV, extravasations, hemorrhage and fibrovascular scar formation occur in the subretinal space, leading to the death of the neurosensory retina and vision loss (Al-Zamil and Yassin, 2017; Hobbs and Pierce, 2021). How does the accumulated drusen induce neovascularization? Although vascular endothelial growth factor (VEGF) has been established as the key factor that initiates and promotes CNV, neuroinflammation also modulates CNV formation (Balser et al., 2019; Uemura et al., 2021). Activated microglia/macrophages release angiogenic and anti-angiogenic factors and work with VEGF to regulate retinal and choroidal angiogenesis (Pollard, 2009; Welser et al., 2010).

Doyle et al. (2012) showed that isolated drusen from AMD donor eyes can activate the NLRP3 inflammasome and secretion of IL-1b and IL-18. In a model of wet AMD with laser-induced CNV, IL-18 activation inhibited CNV development in *Nlrp3*^−/−^ mice (Doyle et al., 2012). This study indicates that NLRP3 and IL-18 play a protective role in angiogenesis. Some *in vitro* studies have suggested that IL-18 may inhibit vascular endothelial cell proliferation (Park et al., 2001; Kim et al., 2005, 2006). The results of numerous studies are consistent with those of Doyle et al. (2012) that IL-18 has an antiangiogenic effect (Kim et al., 2005, 2006). The findings of Qiao et al. support the notion that IL-18 plays a role in suppressing angiogenesis by promoting the regression of pathologic neovascularization (NV), and the work of Cao et al. revealed an inhibitory effect of IL-18 on FGF-induced NV (Cao et al., 1999; Qiao et al., 2007). Therefore, IL-18 neuroinflammation may function in part to repress CNV formation in AMD, though other studies have failed to observe a significant contribution of IL-18 deficiency to spontaneous CNVM formation in *Vegfa*^hyper^ mice (Malsy et al., 2020; Marneros, 2021).

RIPK-dependent microglial necroptosis is also involved in retinal angiogenesis. He et al. argued that microglial necroptosis may be an important biological process in the etiology of retinal angiogenesis. They identified a subpopulation of microglia that highly express RIPK3 and MLKL through single-cell RNA sequencing in an oxygen-induced retinopathy (OIR) model (He et al., 2021). This necroptotic subpopulation of microglia promoted angiogenesis by releasing intracellular FGF2 (an angiogenic factor). The extracellular release of FGF2 was reduced by Nec1 treatment or conditional deletion of *Ripk3*. Moreover, combined treatment with anti-RIPK1 and anti-VEGF drugs has been shown to remarkably suppress angiogenesis in an OIR model (He et al., 2021). Collectively, this evidence reveals a new mechanism by which microglial necroptosis contributes to angiogenic retinopathy.

RIPK also regulates angiogenesis *via* non-necroptotic modulation of macrophages. Ueta et al. demonstrated that RIPK1 inhibition suppressed angiogenesis by modulating macrophage polarization in a mouse model of laser-induced CNV and alkali injury-induced corneal neovascularization (Ueta et al., 2019). Mechanistically, we revealed that RIPK1 inhibition mediates caspase activation and suppresses the pro-angiogenic M2-like phenotype in infiltrating macrophages. Therefore, RIPK may be a potential therapeutic target of pathological angiogenesis that could both inhibit necroptosis and modulate microglia/macrophage polarization.

### Role of RIPK in Glaucoma

Glaucoma is characterized by the progressive loss of retinal ganglion cells (RGCs) and is a leading cause of irreversible blindness (Quigley and Broman, 2006). Glaucomatous neurodegeneration is attributed to various pathologies, such as mechanical pressure, vascular deficiency, inflammation, oxidative stress and metabolic dysregulation (Tezel, 2021). Elevated intraocular pressure (IOP) is the major risk factor for the progression of glaucoma, and pressure-induced retinal ischemia aggravates the death of RGCs (Sellés-Navarro et al., 1996).

While apoptosis has traditionally been considered the major type of RGC death in glaucoma, attempts to rescue RGCs by regulating apoptosis in glaucoma have been unsuccessful. Accumulating evidence indicates that both apoptosis and necroptosis participate in glaucomatous RGC death, and RIPK also plays a crucial role (Lee et al., 2013; Dvoriantchikova et al., 2014; Do et al., 2017; Wang et al., 2020). Dvoriantchikova et al. demonstrated that RIPK inhibition prevented RGC necroptosis in both an *in vitro* and an *in vivo* model of ischemic injury (Dvoriantchikova et al., 2014). Do et al. reported that RIPK1 was activated in a rat model of high IOP-induced ischemic injury, and a novel RIPK1-inhibitory compound significantly attenuated RGC death in a dose-dependent manner (Do et al., 2017). Consistent with these *in vivo* findings, Xiong et al. revealed that RIPK-mediated necroptosis is induced by oxygen-glucose deprivation in retinal ganglion cell line 5 (RGC-5) (Liao et al., 2017). Subsequently, they found that p90 ribosomal protein S6 kinase 3 (RSK3) is an upstream regulator of RIPK3 phosphorylation, and that an RSK3 inhibitor conferred RGC protection and functional recovery in rat with high IOP-induced ischemic injury (Wang et al., 2020). Our previous study also revealed that the expression of RIPK was increased in an optic nerve (ON) crush model, and necrosis inhibitors promoted a moderate level of axonal regeneration (Kayama et al., 2018). Collectively, these findings suggest that RIP kinase-dependent necroptosis is both a novel mechanism of RGC death and a suitable therapeutic target.

As described above, neuroinflammation is one of the pathogenic mechanisms of RGC degeneration in glaucoma (Weinreb et al., 2014). TNF-α is elevated in the aqueous humor, retina and optic nerve of glaucoma patients (Yan et al., 2000; Tezel, 2008; Sawada et al., 2010) as well as in a glaucoma mouse model induced by chronic ocular hypertension (Nakazawa et al., 2006b). In the latter study in mice (Nakazawa et al., 2006b) further showed that increased IOP induces TNF-α production in the retina and optic nerve, and ultimately causes RGC loss *via* microglia activation (Figure 3). Moreover, they found that intravitreal TNF-α injection induced RGC death that mimics that the glaucoma, and blockade of microglial activation rescued this RGC death induced by TNF-α (Nakazawa et al., 2006b). Collectively, these findings of Nakazawa et al. (2006b) highlight the importance of TNF-α and microglial neuroinflammation in the pathology of glaucoma. Other authors similarly showed that microglia are highly activated in the optic nerve head in human eyes with glaucoma, in association with the abundant expression of TNF-α, TGF-β and matrix metalloproteinases (Neufeld, 1999; Yuan and Neufeld, 2001).

The roles of RIPK and necroptosis in the neuroinflammation of glaucoma are still elusive. Dvoriantchikova et al. demonstrated that RIPK inhibition attenuates the expression of pro-inflammatory genes such as Il-1b, Ccl5, Cxcl10, Nos2, and Cybb in a high-IOP induced ischemic injury model, suggesting that RIPK may also mediate neuroinflammation in glaucoma (Dvoriantchikova et al., 2014).

Using a neuroinflammatory model of glaucoma (TNF-α induced RGC degeneration), Ko et al. revealed that a non-canonical form of necroptosis is critical for axonal degeneration of RGCs. They showed that sterile alpha and TIR motif 1 (SARM1), an inducible NAD+ cleavage enzyme, act downstream of TNF-α and activate MLKL. In this model, MLKL does not directly induce necroptosis but rather mediates the loss of axon survival factors NMNAT2 and SCG10/STMN2, which leads to axon degeneration. Therefore, inhibition of TNF-α and/or SARM1 may suppress axon necroptosis, and these pathways may serve as alternative targets for glaucomatous neurodegeneration.

## Prospects for Anti-Ripk/Necroptosis Treatment in Retinal Disorders

RIPK-dependent necroptosis has been proposed as a promising target for a variety of neurodegenerative diseases that involve both neuronal cell death and neuroinflammation. Inhibition of the RIPK pathway has been proven effective in blocking necroptosis and suppressing neuroinflammation in many of the basic experiments mentioned above, and therefore, strategies to translate these studies into clinical trials should be explored.

In preclinical studies, RIPK1 inhibitors have been the agents most studied for the treatment of CNS diseases. Such research has shown that Nec-1 administration controls the neuronal cell death induced by acute neuronal injury and improves motor and spatial memory (You et al., 2008). Nec-1 also promotes the ability of microglia to degrade Aβ in APP/PS1 mice (Ofengeim et al., 2017). The RIPK1 inhibitor GSK′547 ameliorated the aggregation of lipofuscin-like lysosomal inclusions in microglia and improved survival in a model of lysosomal storage disorder (Safaiyan et al., 2016; Cougnoux et al., 2018). RIPK is activated under various necrotic or non-necrotic pathological inflammatory conditions, and thus well-designed drugs that target certain RIPK regulation points are critical for the treatment of human CNS and peripheral pathologies.

Indeed, Nec-1s and other RIPK1 inhibitors (e.g., GSK2982772, DNL104, DNL747, and DNL788) are presently being assessed in clinical trials for neurodegenerative and autoimmune diseases (Degterev et al., 2019; Mifflin et al., 2020). A phase II study of GSK2982772 for the treatment of psoriasis and ulcerative colitis (UC) was completed. The authors reported that GSK2982772 was safe and well-tolerated when dosed at 60 mg t.i.d., and that it may have ameliorated inflammation in 65 psoriasis patients (Weisel et al., 2020). They then assessed the potential effects at higher dosages and in patients with more active disease (Weisel et al., 2020). However, GSK2982772 showed no significant difference in clinical efficacy between the treatment and placebo groups in UC patients (Weisel et al., 2021). A phase I clinical trial of DNL747 for the treatment of AD has been completed, but the results have not been disclosed. RIPK3 inhibitors have not yet entered clinical trials, but studies on these agents are developing rapidly (Jensen et al., 2020).

Although the existing results of these clinical trials show the safety of anti-RIPK drugs with currently unknown efficacy, it will be critical to develop more secure, sustained, and effective inhibitors of RIPK to combat chronic neurodegeneration. Therefore, medicines with better pharmacological designs and sustained local delivery may be required for the further development of anti-RIPK therapy to prevent blindness in patients with currently incurable retinal degeneration.

## Author Contributions

YM proposed and guided the direction of the manuscript. YT and YM wrote the main body of this manuscript. K-HS and DV provided supervision of the review. All authors contributed to the article and approved the submitted version.

## Funding

This work was supported by Grants-in-Aid for Scientific Research, #JP19K09952 and #JP22H03242 (to YM), a Uehara Memorial Foundation grant (to YM), a Charitable Trust Fund for Ophthalmic Research in Commemoration of Santen Pharmaceutical's Founder grant (to YM), and a Bayer Retina Award (to YM).

## Conflict of Interest

The authors declare that the research was conducted in the absence of any commercial or financial relationships that could be construed as a potential conflict of interest.

## Publisher's Note

All claims expressed in this article are solely those of the authors and do not necessarily represent those of their affiliated organizations, or those of the publisher, the editors and the reviewers. Any product that may be evaluated in this article, or claim that may be made by its manufacturer, is not guaranteed or endorsed by the publisher.
